# Supplementary material for: Vitamin D Modified DSS-Induced Colitis in Mice via STING Signaling Pathway
Source: Biology (Basel). 2025 Jun 18;14(6):715. doi: 10.3390/biology14060715 (PMC12190092; doi:10.3390/biology14060715)
Supplement: Supplementary file 1 [file biology-14-00715-s001.zip › Supplementary Materials S1: 16s-report of gut microbiota/Groups/betadiv/pca_analysis/PCA23_boxplot_ellipse.pdf]

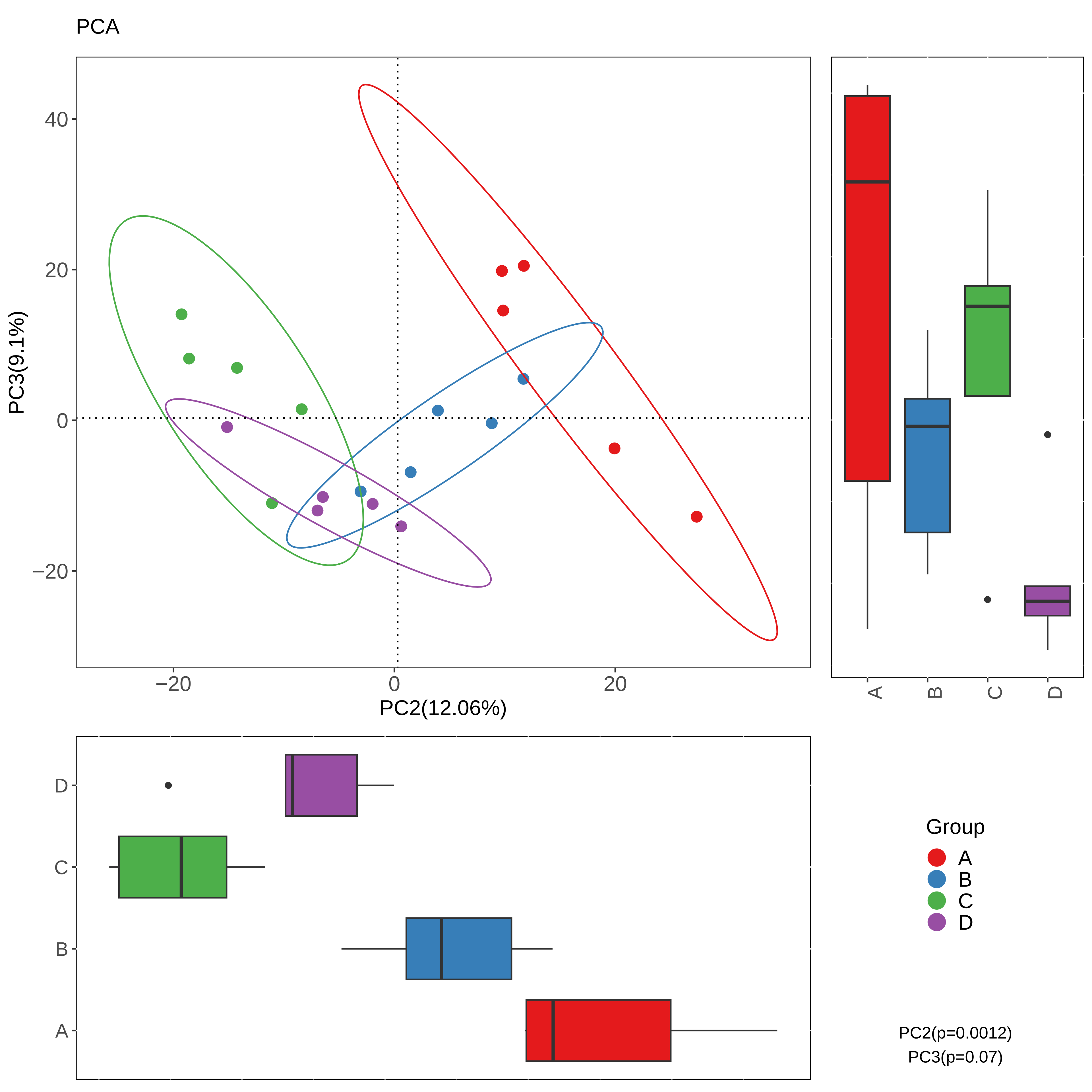

PCA
PC3(9.1%)
PC2(12.06%)
40
20
0
−20
−20
0
20
A
B
C
D
Group
A
B
C
D
PC2(p=0.0012)
PC3(p=0.07)
